# A New Method for Feedback on the Quality of Chest Compressions during Cardiopulmonary Resuscitation

**DOI:** 10.1155/2014/865967

**Published:** 2014-08-28

**Authors:** Digna M. González-Otero, Jesus Ruiz, Sofía Ruiz de Gauna, Unai Irusta, Unai Ayala, Erik Alonso

**Affiliations:** Communications Engineering Department, University of the Basque Country (UPV/EHU), Alameda Urquijo S/N, 48013 Bilbao, Spain

## Abstract

Quality of cardiopulmonary resuscitation (CPR) improves through the use of CPR feedback devices. Most feedback devices integrate the acceleration twice to estimate compression depth. However, they use additional sensors or processing techniques to compensate for large displacement drifts caused by integration. This study introduces an accelerometer-based method that avoids integration by using spectral techniques on short duration acceleration intervals. We used a manikin placed on a hard surface, a sternal triaxial accelerometer, and a photoelectric distance sensor (gold standard). Twenty volunteers provided 60 s of continuous compressions to test various rates (80–140 min^−1^), depths (3–5 cm), and accelerometer misalignment conditions. A total of 320 records with 35312 compressions were analysed. The global root-mean-square errors in rate and depth were below 1.5 min^−1^ and 2 mm for analysis intervals between 2 and 5 s. For 3 s analysis intervals the 95% levels of agreement between the method and the gold standard were within −1.64–1.67 min^−1^ and −1.69–1.72 mm, respectively. Accurate feedback on chest compression rate and depth is feasible applying spectral techniques to the acceleration. The method avoids additional techniques to compensate for the integration displacement drift, improving accuracy, and simplifying current accelerometer-based devices.

## 1. Introduction

Chest compressions delivered at an adequate depth and rate, allowing full chest recoil, and with minimal interruptions are key to improve survival from cardiac arrest [1–3]. Current cardiopulmonary resuscitation (CPR) guidelines [4, 5] recommend chest compression depths and rates of at least 5 cm and 100 min^−1^, respectively. However, out-of-hospital and in-hospital studies on CPR quality show that delivering chest compressions with adequate rate and depth is difficult, even among well-trained responders [6, 7]. The use of real-time CPR feedback devices has contributed to improve the quality of CPR provided by lay people and trained rescuers in both simulated and real life scenarios [8, 9].

The first CPR feedback devices used force/pressure sensors on the assumption of a linear relation between compression force and depth [10–12]. However, the chest has a nonlinear variable stiffness within the compression cycle which varies among individuals [13–16], a fact that has been confirmed on cardiac arrest data with simultaneous force and depth recordings [17]. Consequently, most current CPR feedback devices are based on accelerometers. These devices calculate the instantaneous displacement of the chest, that is, the compression depth (CD) signal, by integrating the acceleration twice [9]. However, noise in the acceleration signal compromises the accuracy of methods based on the double integration. Even a small offset in the acceleration signal produces integration errors that rapidly accumulate, making feedback impossible unless the resulting displacement drift is compensated for every compression [18]. Over the last decade several drift compensation mechanisms have been conceived, giving rise to complex and sometimes bulky devices that incorporate additional sensors [19, 20] and/or use elaborate signal processing techniques [1, 21–23].

Accelerometer-based devices calculate rate and depth values for feedback for each compression [24–26]. Audiovisual feedback to the rescuer is then given at every compression or by averaging these values over the last 3–5 compressions [24, 25]. Feedback on rate and depth at every compression seems excessive and may be ignored by the rescuer [9, 27]. A more sensible approach to feedback would be to average rate and depth over the last compressions, resulting in feedback times somewhere in the 2–5 s range.

This study introduces a new paradigm on accelerometer-based devices. Instead of calculating the CD signal, feedback on the average rate and depth during a short analysis interval is directly computed from the acceleration by means of spectral techniques. Drift compensation or additional sensors would no longer be needed, giving rise to simpler, smaller, and more user-friendly feedback devices.

## 2. Materials and Methods

### 2.1. Equipment and Data Collection

**Figure 1:**
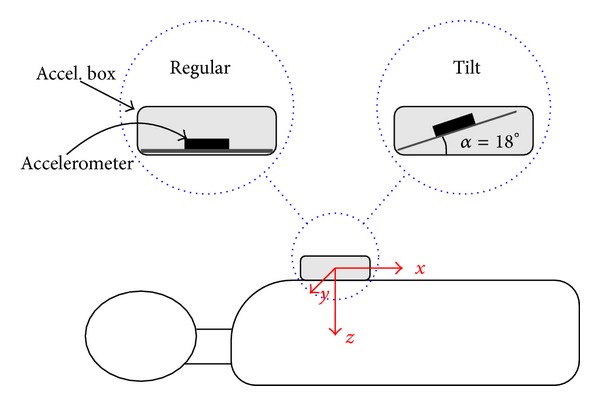
Positioning of the accelerometer within the enclosure for the regular and tilt sessions. During each recording the enclosure was kept fixed to the manikin's chest.

A Resusci Anne manikin (Laerdal Medical, Norway) was equipped with a photoelectric sensor (BOD 6K-RA01-C-02, Balluff, USA) to register the actual CD signal, which was used as gold standard. The accelerometer (ADXL330, Analog Devices, USA) was placed in an enclosure which was fixed to the manikin's sternum, and the manikin was placed on the floor, as shown in Figure 1. The three acceleration axes and the CD signal were digitized using an NI-USB6211 (National Instruments) data acquisition card with a sampling rate of 500 Hz and 16-bit resolution.

Twenty volunteers received basic compression-only CPR training before participating in two recording sessions: a regular session, in which the vertical axis of the accelerometer was perpendicular to the manikin's chest, and a tilt session, with an 18° misalignment (see Figure 1). These sessions were defined to study situations in which the accelerometer may not be in a fixed position relative to the patient's chest. In each session the volunteers delivered 60 s of uninterrupted compressions eight times, combining different target rates (80, 100, 120, and 140 min^−1^) and depths (30 mm and 50 mm). A metronome was used to guide compression rate, and a custom-made computer program displayed the CD signal in real-time to guide compression depth.

The recorded signals were preprocessed with a third-order Butterworth low-pass filter (cut-off frequency 15 Hz) to suppress high-frequency noise and resampled to 100 Hz. Compressions were automatically identified in the CD signal using a peak detector with a fixed 15 mm threshold, and the annotations were then manually reviewed.

### 2.2. Feedback on Rate and Depth

#### 2.2.1. Mathematical Model

Feedback was calculated for short analysis intervals during continuous chest compressions. If the intervals are short, then it is possible to assume that all chest compressions within the analysis interval are very similar. Mathematically this means that acceleration and CD are almost periodic signals, whose fundamental frequency is the mean frequency of the compressions, *f*_cc_ (Hz). For each analysis interval, their periodic representation, denoted by *a*(*t*) for the acceleration and *s*(*t*) for the CD signal, is then a good approximation of the real signals. These periodic representations can be modelled using the first *N* harmonics of their Fourier series decomposition (without DC component):

(1) a(t)=∑k=1NAkcos⁡⁡(2πkfcct+θk) (m/s2),

(2) s(t)=∑k=1NSkcos⁡(2πkfcct+ϕk) (mm).

Since the feedback device records the acceleration the problem is then to obtain *s*(*t*) from *a*(*t*) knowing that the acceleration and the displacement are related by

(3) $$\begin{array}{c} a\left(t\right)=\frac{d^{2}s\left(t\right)}{dt^{2}} \end{array}$$

which, in the general case, involves a double integration of the acceleration signal. However, for the quasiperiodic approximation, using the Fourier series representation of *a*(*t*) and *s*(*t*) in (3) yields the following relations between the amplitudes and phases of their harmonics:

(4) $$\begin{array}{c} S_{k}=\frac{1000A_{k}}{{\left(2\pi kf_{\text{cc}}\right)}^{2}},\;\;\phi_{k}=\theta_{k}+\pi ,\;\text{for}\;\;k=1,2,\ldots ,N. \end{array}$$

These equations can be used to reconstruct *s*(*t*) once *f*_cc_, *A*_*k*_, and *θ*_*k*_ are obtained from the acceleration signal.

#### 2.2.2. Spectral Method for Feedback on Rate and Depth

**Figure 2:**
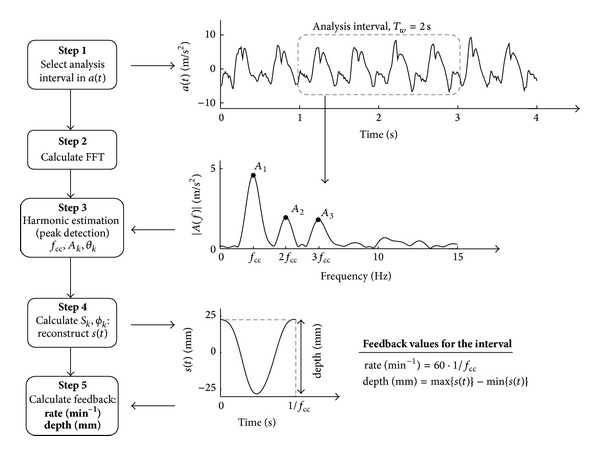
Block diagram of the spectral method for feedback on rate and depth.

Spectral analysis was used to estimate the harmonics of *a*(*t*) needed to reconstruct *s*(*t*). In summary, feedback on the mean rate and depth for each analysis interval were obtained following the steps described in Figure 2. In Step 1, a Hamming window was applied to the acceleration signal to select the analysis interval. Its 2048-point fast Fourier transform (FFT) with zero padding was computed in Step 2. Then, the first three harmonics and their fundamental frequency were estimated (Step 3). Equation (4) was used to compute *S*_*k*_ and *ϕ*_*k*_, which were used to reconstruct *s*(*t*) from (2) (Step 4). Finally, in Step 5, feedback on rate and depth were obtained using the reconstructed cycle of *s*(*t*) as

(5) rate (min⁡−1)=60·fcc,depth (mm)=max⁡{s(t)}−min⁡{s(t)}.

Several characteristics of the method such as the Hamming window, the number of harmonics, and the number of points to compute the FFT were selected using signal processing criteria to guarantee a high accuracy.

### 2.3. Performance Evaluation

To evaluate the accuracy of the method we assumed feedback would be given at the end of each analysis interval; consequently records were divided into nonoverlapping consecutive analysis intervals of duration *T*_*w*_. For each analysis interval, feedback for rate and depth obtained by the method was compared to that obtained from the distance sensor placed inside the manikin.

First, the mean rate and depth per record were analysed for the different targeted CPR test conditions. The distributions of the mean rate and depth did not pass the Kolmogorov-Smirnov normality test and are presented as median (5–95 percentiles). The median values obtained from the gold standard and the method were compared using the Mann-Whitney *U* test, and differences were considered significant for *P* values under 0.05. Then, errors in rate/depth feedback were obtained for every analysis window. The root-mean-square error (RMSE) of all feedbacks in a session (regular/tilt) was used to measure the global accuracy of the method as a function of the duration of the analysis interval, *T*_*w*_. Finally, a Bland-Altman analysis [28, 29] was conducted for *T*_*w*_ = 3 s to assess the agreement on feedback between the gold standard and the method, and the 95% limits of agreement (LOA) were obtained.

## 3. Results

**Figure 3:**
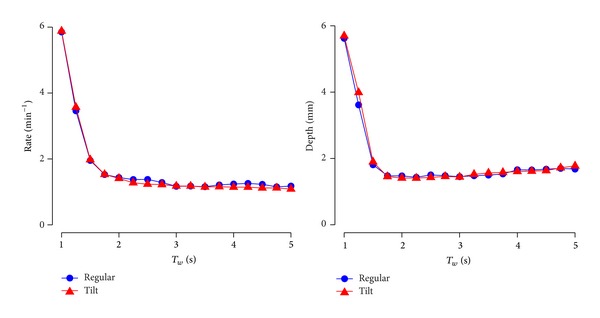
Root-mean-square error (RMSE) in mean rate and depth as a function of the duration of the analysis interval for the regular session and the tilt session.

**Figure 4:**
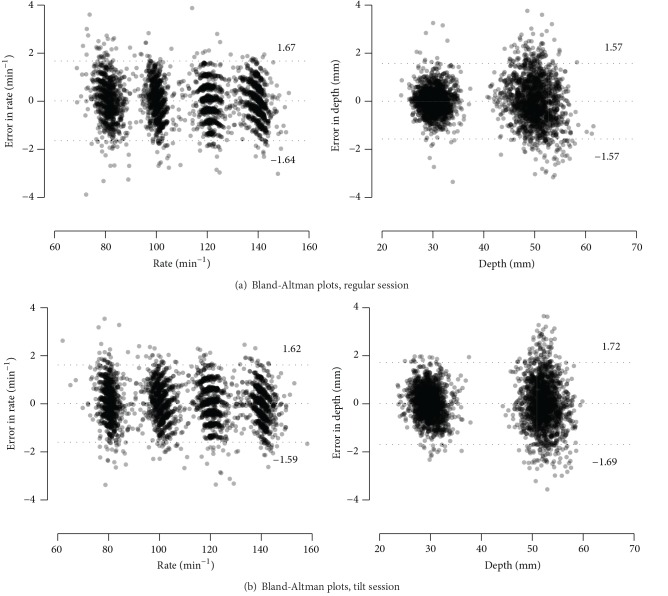
Bland-Altman plots of the errors plotted against the gold standard (from the photoelectric sensor), for the regular (a) and tilt (b) sessions. The 95% levels of agreement are indicated in text and by horizontal lines.

The dataset comprised 320 60 s records with a total of 35 312 compressions.  Table 1 compares the mean rate and depth per episode obtained from the gold standard and the method when *T*_*w*_ = 3 s. There was no significant difference between the method and the gold standard for any of the CPR target conditions.  Figure 3 shows the RMSE as a function of *T*_*w*_ for the tilt and regular sessions. For *T*_*w*_ between 2 and 5 s the RMSE for rate and depth were below 1.5 min^−1^ and 2 mm. Finally, Figure 4 shows the Bland-Altman plots of the difference between the method and the gold standard for *T*_*w*_ = 3 s. For the regular session, the differences in feedback for rate and depth showed a 95% LOA of −1.64–1.67 min^−1^ and −1.57–1.57 mm, respectively. For the tilt session, the differences in feedback for rate and depth showed a 95% LOA of −1.59–1.61 min^−1^ and −1.69–1.72 mm, respectively.

## 4. Discussion

CPR feedback on chest compression rate and depth improves the quality of CPR both during training [25, 30] and in the field [27, 31, 32]. Currently, most real-time devices for CPR feedback are based on the double integration of the acceleration which inevitably requires adding drift compensation techniques [18, 33] that result in bulky devices and/or occasional inaccurate depth feedback [19, 34]. This study presents, to the best of our knowledge, the first accelerometer-based method for feedback on rate and depth of chest compressions that avoids the drift problem. The method is based on simple and optimised spectral techniques making it computationally very efficient. These considerations would utterly simplify current accelerometer-based devices.

The accuracy of the method was tested in a manikin platform. This allowed the recording of the actual instantaneous chest compression depth for use as gold standard but also the testing the algorithm for a wide range of controlled conditions: different rescuers, target depths and rates, and the influence of the relative position of the device and the chest (regular versus tilt). Misalignment between the device and the chest was tested for two reasons. First, although the device is usually in contact with the patient's chest, the sternum may not be completely horizontal due to anatomical considerations, even when the patient is in supine position. Second, other suitable positions of the device could be envisioned, such as on top of the hand or fixed to the wrist. In those situations tilt may vary during chest compressions. In either case, there were no significant differences in rate and depth feedback between the gold standard and the method. Moreover, for all the tested conditions the RMSE for rate and depth were below 1.5 min⁡^−1^ and 2 mm, respectively, which guarantees a very accurate feedback for analysis intervals in the 2–5 s range. Furthermore, the Bland-Altman analysis revealed that all individual feedbacks were very accurate and that the method is reliable because it did not present outliers.

The method presented in this study directly estimates the mean rate and depth for feedback without the need to obtain the instantaneous CD signal. This avoids the need to integrate the acceleration signal twice. Double integration introduces a large displacement drift [18], which has to be compensated. Over the years several techniques have been developed to correct the displacement drift. Some solutions correct the drift for each compression cycle. This involves the detection of the start of each compression using either additional force sensors [18, 20] or a combined analysis of the CD and the ECG signals [21]. Others compensate the drift adaptively using filters based on additional reference signals such as force, blood pressure, ECG, or thoracic impedance [19]. However, incorporating additional sensors makes the feedback device more complex, and recording the ECG bounds the feedback device to the defibrillator. Alternatively, solutions based exclusively on signal processing techniques have also been developed to minimize or cancel the drift [1, 22, 35]. However, these techniques may introduce errors in depth as large as 6 mm for 95% of the cases [24]. The spectral technique introduced in this paper is more robust to acceleration noise because it only estimates three harmonic components of the acceleration for an accurate feedback.

Improvement of CPR quality relies on two key factors: real-time monitoring of CPR parameters and debriefing [36–38]. Real-time feedback in short time intervals is demonstrated in this study. Debriefing could easily be implemented simply by storing the rate and depth feedback values for each interval. These values could then be used to obtain postresuscitation scorecard with global measures of CPR quality and graphs of the time evolution of rate and depth [39].

The method shares two common limitations of all accelerometer-based devices. First, accurate depth feedback is compromised if there is incomplete chest recoil, that is, rescuer leaning [38]. The actual depth of a compression is the displacement of the patient's sternum from its resting position towards the spine. Accelerometer-based devices are accurate only if the sternum returns to its resting position on every compression [23]. Otherwise, the only solution is to detect incomplete chest recoil and then launch an alarm to correct excessive leaning [38]. Second, the study was conducted for the manikin resting on a hard incompressible surface. On softer surfaces depth is overestimated as the sum of the sternum-spine displacement and mattress compression [40]. This drawback can be corrected by using two aligned accelerometers (chest and back) and processing the difference of the recorded accelerations [26, 35]. Our method can be directly adapted to use the difference of the two accelerometers.

We demonstrated the accuracy of the method during continuous chest compressions. However, a full evaluation of the method using retrospective out-of-hospital cardiac arrest records in which acceleration signal is available is still needed. Such study would serve to evaluate the feasibility and reliability of the method in real resuscitation scenarios, where pauses in chest compressions are frequent and the acceleration patterns may differ from those generated in manikins.

## 5. Conclusion

This study introduces a new paradigm in accelerometer-based CPR feedback devices because it allows calculating rate and depth values for feedback without reconstructing the instantaneous CD signal. It avoids additional techniques to compensate the drift caused by accelerometer noise and double integration, thus simplifying feedback devices. Feedback is accurate for analysis intervals of a few seconds during continuous chest compressions. Further studies with retrospective episodes would serve to evaluate the feasibility and reliability of the method in a real resuscitation scenario.

## Figures and Tables

**Table 1:** Median values (5/95 percentile in parenthesis) of the mean rate and depth per record for the regular and tilt sessions with *T*_*w*_ = 3 s. The Mann-Whitney *U* test was used to compare the values obtained from the gold standard (CD signal) and the acceleration (accel. signal). No significant difference was observed for any of the CPR target conditions.

Target	Regular session		Tilt session	
	CD signal	Accel. signal	CD signal	Accel. signal
Rate^a^				
80 min^−1^	80.8 (77.4–84.8)	80.9 (77.5–84.9)	80.2 (77.5–83.6)	80.3 (77.7–83.6)
100 min^−1^	100.1 (97.5–102.2)	100.0 (97.4–102.3)	100.3 (96.5–105.1)	100.1 (96.2–105.3)
120 min^−1^	120.5 (117.4–123.8)	120.4 (117.6–123.9)	120.2 (116.9–123.7)	120.3 (116.9–124.0)
140 min^−1^	140.1 (134.4–142.0)	140.2 (134.8–142.1)	140.2 (135.1–143.4)	140.1 (135.3–143.2)
Depth^b^				
30 mm	30.4 (27.7–33.3)	30.3 (27.0–33.5)	29.7 (27.5–32.6)	29.7 (27.0–33.2)
50 mm	50.1 (45.4–54.1)	50.1 (45.6–54.9)	52.3 (49.1–55.1)	52.5 (49.1–57.0)

^a^40 records per session, ^b^80 records per session.
